# Psychosocial study environment characteristics associated with exposure to sexual harassment at a large public university in southern Sweden: a cross-sectional study

**DOI:** 10.1080/16549716.2023.2264627

**Published:** 2023-10-12

**Authors:** Jack Palmieri, Per-Olof Östergren, Markus Larsson, Anette Agardh

**Affiliations:** Social Medicine and Global Health, Department of Clinical Sciences, Lund University, Malmö, Sweden

**Keywords:** Sexual harassment, psychosocial study environment, demand-control-support, university students, study strain

## Abstract

**Background:**

Universities can be understood as work-like environments for students, with similar risks and expectations regarding psychosocial environment. Limited research has examined this study environment from a Demand-Control-Support perspective with regard to sexual harassment. Understanding this environment is key to designing protective measures. This study aimed to examine the association between individual and psychosocial study environment characteristics and exposure to sexual harassment among students at Lund University, Sweden.

**Methods:**

This cross-sectional study utilised data from an online survey conducted among students. Questions on background characteristics, exposure to sexual harassment while at university and psychosocial study environment as measured by a Demand-Control-Support-instrument were used. Bivariate, and multivariable logistic regressions were used, together with Population Attributable Fractions (PAF), and synergy indexes (SI).

**Results:**

High demands and low control were independently associated with higher odds of being exposed to sexual harassment among both females and males (OR 1.41, OR 1.26 and OR 1.55, OR1.34, respectively). When adjusting for background characteristics, high study strain (combination of high demands and low control) was associated with exposure to sexual harassment among both female and male respondents (aOR 1.67 and 1.98 respectively) and could account for PAF of 14% and 15% of study environment sexual harassment for females and males, respectively. Low lecturer support was associated with higher odds for sexual harassment among females (aOR 1.19) but not males. Little evidence was found for a buffering effect of student support on high strain and sexual harassment (SI 0.7).

**Conclusion:**

Working to reduce situations of high strain study environments could be an effective strategy for reducing sexual harassment in university settings. Improving support from lecturers could also modify this relationship, but more research is required to identify causal pathways underlying this result.

## Introduction

Several decades worth of research concerning workplace sexual harassment (SH) have shown it to be a pernicious and prevalent problem in most organisations and institutions. Although prevalence data can be difficult to compare, in one of the few national studies about the issue, Rospenda, Richman and Shannon concluded that one in two women in the USA had been exposed to sexual harassment in the previous year [1]. In research conducted in Europe, these statistics varied between 17 and 81% of women [2].

Workplace SH has been associated with a host of negative outcomes for individuals and organisations including adverse psychological health outcomes [3,4] and physical manifestations [5] for the individual, and far-reaching job-related outcomes including withdrawal, loss of productivity, and lowered job satisfaction [5]. A recent cohort study conducted in Sweden showed a prospective association between exposure to workplace sexual harassment and suicidal behaviour [6].

Understanding which factors are associated with exposure to sexual harassment, both on an individual level, as well as on an organisational level, is key to preventative work against sexual harassment.

Universities and other sites of higher education are complex spaces of work, study, and social interaction. A recent systematic review of the literature highlights the prevalence of SH in this context and suggested that one in four female students had been exposed to sexual harassment at university [7], while a large Norwegian study presented figures of 21.6% among women, and 5.7% among men regarding exposure to SH in the past year [8]. In a study conducted at Lund University, 26.8% of female students, and 11.3% of male students reported having been exposed to SH [9]. Consequences of exposure to SH among students include physical symptoms such as pain, increased alcohol use, and post-traumatic stress disorder [7]. Less evidence is available regarding the impact of SH on academic performance [7].

Many different factors can intersect as predictors of SH but also affect how such harassment is perceived and how the outcomes can be experienced [5]. Research into antecedent factors associated with workplace SH suggests that organisational climate (tolerance for sexual harassment) is the single best predictor of SH [10], while other studies have examined the relationship between job strain and exposure to sexual harassment [6].

Despite this, little research has been conducted that examines the study environment of students using the Demand-Control-Support framework (DCS) [11]. The Demand-Control-Support model postulates that work/study stress primarily comes from the interaction between psychological demands due to work and the effect of lack of autonomy that allows employees to make their own decisions, commonly labelled lack of ‘control’ [12]. It also acknowledges the importance of social support both as a potential buffer or, in its absence, as an additional stressor.

Studies utilising DCS in university settings tend to focus on internships and work placements that resemble more a traditional workplace [13] or are smaller experimental studies that do not address the study environment as a whole [14]. Moreover, they tend to focus on the associations between stress and academic performance [15], stress and wellbeing [16,17], burnout [18], and intention to leave studies [13]. No previous studies have examined the associations between DCS and sexual harassment among university students.

Drawing clear distinctions between SH and other forms of mistreatment, harassment, and incivility can be complex as there are myriad definitions and instruments used to research these issues. Research outside of an academic setting into DCS and other forms of harassment, bullying or incivility is often guided by the ‘work environment hypothesis’ that posits that ‘stressful and poorly organised work environments may give rise to conditions that may develop into bullying’ [19]. In this context, research has shown a positive association between high demand and low control and bullying among police officers in Australia [20], blue collar workers in Belgium and Spain [21], and diverse workers from the USA [22]. Not all research has shown such results, and in a study conducted among nurses and midwives in Australia, the DCS variables did not predict bullying, although high demands did predict external threat of assault and external emotional abuse [23]. In much of this research, it is specifically the combination of high demands and low control (defined as high strain) that is shown to make employees vulnerable to workplace bullying [24].

In order to better understand the study environment context and whether it is associated with sexual harassment, the aim of this study was to examine individual and study environment characteristics associated with exposure to sexual harassment among students at Lund University, Sweden.

## Methods

### Study setting and data collection

Lund University is a public university in southern Sweden with around 31,000 students and 8,000 staff. In November 2019, all students enrolled in undergraduate and graduate courses were invited to participate in a survey as part of the ‘Tellus’ project. The ‘Tellus’ project is a university-based initiative aimed at strengthening prevention and response to sexual harassment.

The survey instrument was self-administered online, and contained sections on background, study environment and sexual harassment, as well as health and social capital. Participants could answer the questionnaire in English or in Swedish, and all submissions were anonymous. Students who chose to participate were given a cinema ticket as compensation for their time utilising a system that allows emails to be sent to participants without connecting that participant to any submitted survey. Ethical approval was received from the Swedish Ethical Review Authority (number: 2018/350).

### Study measures

#### Outcome variable

*Sexual harassment* was defined as conduct of a sexual nature that violates someone’s dignity through, for example, comments or words, groping or indiscreet looks, unwelcome compliments, invitations, or suggestive acts. These definitions were developed from the Swedish discrimination act [25] and the law concerning volunteerism [26].

Previous research has shown that asking respondents to select from a list of behaviours/situations believed to constitute sexual harassment tends to produce higher estimations than asking a single question [3]. Based on this, having experienced *sexual harassment* or *sexual violence* was defined through 10 situations/events adapted from a study conducted among medical students in Canada [27]. For a full discussion of this instrument, and its validation, see Östergren et al. [28].

The final instrument contained the options: Unwelcome suggestive looks or gestures, Unwelcome soliciting or pressuring for ‘dates’, Unwelcome ‘inadvertent’ brushing or touching, Unwelcome bodily contact such as grabbing or fondling, Unwelcome gifts, Unwelcome comments, Unwelcome contact by post or telephone, Unwelcome contact online for example social media or email, Stalking and Attempts to conduct or the conduct of oral, vaginal or anal sex or other equivalent sexual activity in which you did not participate voluntarily. For each option, participants could select ‘Yes, once’, ‘Yes, more than once’ or ‘No’. If they selected ‘Yes, once’, or ‘Yes, more than once’ they were asked to specify when this took place: ‘more than three years ago’, ‘between one and three years ago’ or ‘In the last 12 months’.

For this study, an aggregate variable was created of persons who had reported at least one experience of at least one of the forms of sexual harassment and/or violence listed, at any of the given time points. Such persons were designated as ‘exposed’ and all others as ‘not exposed’.

## Background variables

*Gender Identity* was assessed using two questions, ‘What gender were you assigned at birth’, and ‘What is your current gender identity’. Self-determined gender (question 2) was used where provided, with gender at birth used where not. The second question had three options, female, male, and I do not identify as female or male.

*Age* was recorded as ‘18–25’, ‘26–30’, ‘31–40’ and ‘41 years or older’, then dichotomised as ‘18–25’ and ‘26 and over’.

*Country of birth* was assessed as ‘Sweden’, ‘In a Nordic Country (not Sweden)’, ‘Europe (not a Nordic country)’ or ‘Outside of Europe’.

*Parents born in Sweden* was assessed as ‘Yes, both parents born in Sweden’, ‘No, one parent born outside of Sweden’ and ‘No, both parents born outside of Sweden’. This variable was dichotomised as ‘At least one parent born in Sweden’ and ‘Both parents born outside of Sweden’.

*International student* was assessed through the single question ‘Are you an international student who came to Sweden to study at Lund University?’.

*Study pace* was defined as ‘Full time (100%)’, ‘Part time (50% or more)’ and ‘Part time (less than 50%)’. In this paper, study pace was dichotomised to ‘Full time’ and ‘Part time’.

*Semesters studied at Lund University* were recorded using the following question ‘How many semesters have you studied at Lund University in total? (Including the current semester)’, with options ‘0–1’, ‘2–3’, ‘4–5’, ‘6–7’, ‘8–9’, ’10–11’ and ‘More than 11’. Answers were dichotomised as ‘0–1’ and ‘Greater or equal to 2’.

## Psychosocial study environment

**Figure 1.**
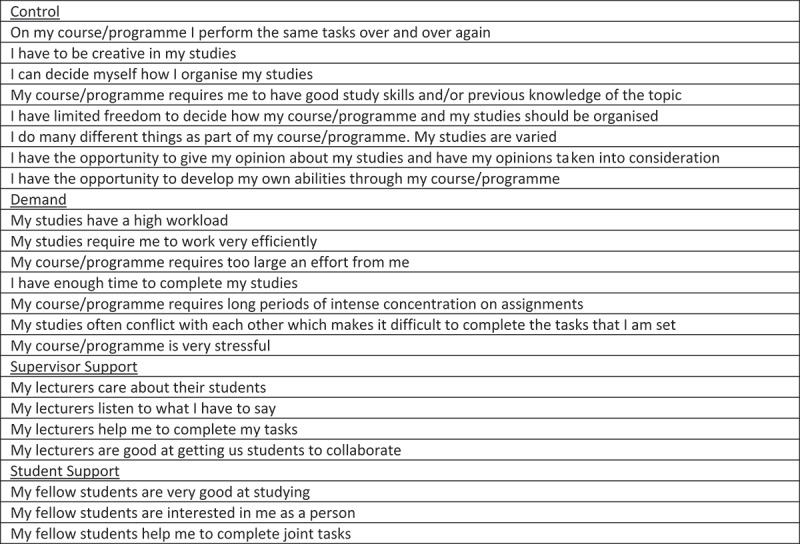
Modified 22-item demand-control-support instrument (English version) for measuring psychosocial study environment.

*Study environment* was assessed by the Demand-Control-Support model [11] operationalised in a modified instrument adapted for the context of heterogeneous university students and validated for dimensionality and internal consistency [29]. The final instrument contains measures for Demands (7 items), Control (8 items), and Supervisor and Student Support (4 and 3 items respectively). Each item was answered on a 4-point score. A short version of these items is presented in Figure 1.

To create the ‘Demand’ and ‘Control’ variables, the scores of all unweighted items in these scales were summed and dichotomised along the median value (cut-off values of 18 and 23 respectively). Study strain was then calculated as follows: ‘high demands’ and ‘low control’ = ‘High Strain study environment’, ‘high demands’ and’ high control’ = ‘Active Study environment’, ‘low demands’ and ‘low control’= ‘Passive Study environment’, and ‘low demands’ and ‘high control’ = ‘Comfortable study environment’.

*Supervisor/Lecturer support* was assessed using 4 items with a 4-point score for each item. These items were summed and dichotomised such that the upper quartile of the summed scores became ‘High support’ and all others became ‘Low support’ (cut-off value 13).

*Student support* was recorded with 3 items, each with a 4-point score. These items were summed and dichotomised at the upper quartile as with supervisor support (cut-off value 10).

## Statistical analysis

Statistical analysis was done using Stata MP Version 16 [30]. Bivariate logistic regressions were used to present an overview of the data. Based on the original theoretical assumption of synergy between high demands and low control and the empirical finding that vulnerability to bullying and harassment is found in such situations [24], study strain was selected as the exposure variable for the multivariable logistic regression. Three models were examined in the logistic regression: model 1 adjusted for age, model 2 further adjusted for status as international student and parents’ country of birth, and model three further adjusted for support from lecturers and students. The decision to control for status as international student and parents’ country of birth is based on the hypothesis that a connection to Sweden is a factor that affects study environment and sexual harassment.

The importance of social support for the relationship between demand and control, as both a potential buffer and additional stressor, is established in existing research [11]. Thus, in the third model, support was also controlled for. These analyses are presented as crude odds ratios (OR) and adjusted odds ratios (aOR) with 95% confidence intervals (CI).

To explore the proportion of exposure to sexual harassment that could be prevented by eliminating the high strain psychosocial work environment, the population attributable fraction was calculated using Miettinen’s formula [31].

Social support has been viewed as a possible modifier of the relationship between psychological workplace strain and health outcomes [32]. The role of support from supervisors/lecturers and students was examined through its main effect in the multivariable logistic regression, through its interaction with job strain and through examining synergy indexes according to the method suggested by Rothman [33].

Previous research has shown gender differences between experiences and consequences of sexual harassment [3]. Due to this, analyses were stratified by gender where appropriate.

## Results

Of 31,064 invitees, a total of 9,787 individuals participated in the study, representing a response rate of 32%. Study respondents who lacked data on both sex and gender (*N* = 46), those who did not answer any of the 10 questions on sexual harassment (*N* = 74) and those who did not answer the questions in the modified DCS-instrument battery (*N* = 707) were excluded. This gave a final study population of 8960.

Of these respondents, a majority identified themselves as female (63%) were under 26 years of age (78%), and born in Sweden (80%), or had at least one Swedish-born parent (77%). Most students studied full time (95%) and had been at the university for at least 2 semesters (70%). Most socio-demographic characteristics were comparably distributed between men and women. Exposure to sexual harassment showed differences, however, where 21% of all respondents reported that they had been exposed to sexual harassment in conjunction with their studies at Lund University, but a much greater proportion was reported among women (27%) than men (12%). Respondents who identified neither as female nor male accounted for under 1% of respondents but reported the highest percentage of exposure to sexual harassment. Due to the small size of this group (*N* = 63) they were excluded from the further analyses in this paper. Table 1 shows the sociodemographic characteristics as well as information on the psychosocial study environment for the respondents in total and stratified by gender.

**Table 1.** Sociodemographic factors, demand, control, study strain, supervisor support and experience of sexual harassment among a sample of Lund University students, total, and stratified by gender.

	All		Female		Male		Neither	
Variables	n	%	n	%	n	%	n	%
**Gender**								
Female	5596	62.5						
Male	3301	36.8						
Neither male nor female	63	0.7						
**Exposed to sexual harassment**								
Yes	1890	21.1	1490	26.6	380	11.5	20	31.8
No	7070	78.9	4106	73.4	2921	88.5	43	62.3
**Age**								
18–25	6966	77.8	4347	77.7	2574	78.0	45	71.4
≥26	1994	22.3	1249	22.3	727	22.0	18	28.6
**International student**								
Yes	1069	12.0	703	12.6	359	10.9	7	11.1
No	7875	88.0	4882	87.4	2937	89.1	56	88.9
* (Missing)*	*(16)*		*(11)*		*(5)*			
**Country of birth**								
Sweden	7161	80.0	4446	79.5	2673	81.0	42	66.7
Nordic country (not Sweden)	209	2.3	147	2.6	59	1.8	3	4.8
Europe (not Nordic)	769	8.6	496	8.9	261	7.9	12	19.0
Outside Europe	816	9.1	504	9.0	306	9.3	6	9.5
* (Missing)*	*(5)*		*(3)*		*(2)*			
**Parents country of birth**								
At least 1 parent born in Sweden	6916	77.2	4305	77.0	2565	77.7	46	73.0
Both parents born outside of Sweden	2042	22.8	1289	23.0	736	22.3	17	27.0
* (Missing)*	*(2)*		*(2)*					
**Semesters studied at Lund University**								
0–1	2721	30.4	1734	31.0	968	29.4	19	30.2
≥2	6236	69.6	3862	69.0	2330	70.7	44	69.8
* (Missing)*	*(3)*				*(3)*			
**Study Pace**								
Full time	8468	94.7	5283	94.6	3128	94.8	57	90.5
Part time	477	5.3	300	5.4	171	5.2	6	9.5
* (Missing)*	*(15)*		*(13)*		*(2)*			
**Control**								
High	4114	45.9	2445	43.7	1646	49.9	23	36.5
Low	4864	54.1	3151	56.3	1655	50.1	40	63.5
**Demand**								
High	4776	53.2	3109	55.6	1619	49.1	38	60.3
Low	4194	46.8	2487	44.4	1682	51.0	25	39.7
**Study strain**								
High strain study environment (high demands low control)	2618	29.2	1766	31.6	829	25.1	23	36.5
Active study environment (high demands high control)	2148	24.0	1343	24.0	790	23.9	15	23.8
Passive study environment (low demands low control	2228	24.9	1385	24.8	826	25.0	17	27.0
Comfortable study environment (low demands high control)	1966	22.0	1102	19.7	856	25.9	8	12.7
**Supervisor support**								
High	2398	26.7	1419	25.4	961	29.1	18	28.6
Low	6562	73.2	4177	74.6	2340	70.9	45	71.4
**Student support**								
High	3393	37.9	2120	37.9	1254	38.0	19	30.2
Low	5567	62.1	3476	62.1	2047	62.0	44	69.8

Table 2 presents the results of the bivariate logistic regression analysis between the various individual (socio-demographic) variables, demand, control, and study strain factors, support from teachers and fellow students, respectively, and exposure to sexual harassment, stratified by gender.

**Table 2.** Bivariate associations between socio-demographic and other factors, including demand, control, and study strain, and exposure to sexual harassment at Lund University, stratified by gender. Odds ratios (OR) and 95% confidence intervals (CI).

Variables	All		Female		Male	
	OR (95% CI)	p	OR (95% CI)	p	OR (95% CI)	p
**Gender**						
Female	**2.79 (2.47–3.15)**	.000				
Male	1 (Ref)					
**Age**						
18–25	**1.69 (1.47–1.93)**	.000	**1.97 (1.68–2.31)**	.000	1.13 (0.86–1.47)	.379
≥26	1 (Ref)					
**International student**						
No	**1.26 (1.07–1.49)**	.006	**1.32 (1.10–1.60)**	.003	1.28 (0.88–1.84)	.197
Yes	1 (Ref)					
**Country of birth**						
Sweden	**1.46 (1.20–1.77)**	.000	**1.48 (1.18–1.85)**	.001	1.44 (0.95–2.19)	.086
Nordic country (not Sweden)	1.03 (0.68–1.56)	.874	0.99 (0.63–1.56)	.952	0.78 (0.26–2.33)	.661
Europe (not Nordic)	**1.33 (1.02–1.72)**	.033	1.21 (0.90–1.64)	.203	1.61 (0.94–2.77)	.083
Outside Europe	1 (Ref)					
**Parents country of birth**						
≥1 parent born in Sweden	**1.27 (1.12–1.45)**	.00	**1.41 (1.21–1.63)**	.000	1.00 (0.77–1.29)	.971
Both parents born outside of Sweden	1 (Ref)					
**Semesters studied at Lund University**						
0–1	1 (Ref)					
≥2	**1.96 (1.73–2.21)**	.000	**2.14 (1.86–2.47)**	.000	**1.69 (1.30–2.20)**	.000
**Study pace**						
Full time	**1.70 (1.30–2.22)**	.000	**1.87 (1.37–2.54)**	.000	1.37 (0.80–2.36)	.250
Part time	1 (Ref)					
**Control**						
Low	**1.34 (1.21–1.49)**	.000	**1.26 (1.12–1.42)**	.000	**1.34 (1.08–1.66)**	.008
High	1 (Ref)					
**Demand**						
High	**1.51 (1.36–1.68)**	.000	**1.41(1.25–1.60)**	.000	**1.55 (1.25–1.93)**	.000
Low	1 (Ref)					
**Study strain**						
High strain study environment (high demands low control)	**2.00 (1.72–2.32)**	.000	**1.76 (1.48–2.10)**	.000	**2.06 (1.51–2.81)**	.000
Active study environment (high demands high control)	**1.49 (1.27–1.76)**	.000	**1.37 (1.13–1.66)**	.001	**1.58 (1.14–2.19)**	.006
Passive study environment (low demands low control	**1.32 (1.12–1.56)**	.001	**1.22 (1.01–1.48)**	.038	1.36 (0.98–1.89)	.068
Comfortable study environment (low demands high control)	1 (Ref)					
**Supervisor support**						
Low	**1.38 (1.22–1.56)**	.000	**1.36 (1.18–1.56)**	.000	**1.28 (1.00–1.64)**	.046
High	1 (Ref)					
**Student support**						
Low	0.90 (0.81–1.00)	.055	0.89 (0.79–1.01)	.066	0.92 (0.74–1.14)	.455
High	1 (Ref)					

Bold font indicates statistical significance.

### Individual background characteristics

Overall, the unadjusted odds of being exposed to sexual harassment were significantly greater among females compared to males (OR 2.79 CI 2.47–3.15). Among females, being in the younger age group, being a non-international student, being Swedish born, and having at least one parent born in Sweden were all significantly associated with higher odds of exposure to sexual harassment than their reference groups. Similar patterns were observed among male respondents, although many of these associations were non-significant.

### Psychosocial study environment

Experiencing low control over one’s studies was significantly associated with being exposed to sexual harassment for both females and males (OR 1.26, CI 1.12–1.42, and OR 1.34 CI 1.08–1.66, respectively). Similarly, experiencing high demands in one’s studies was associated with higher odds of exposure to sexual harassment than experiencing low demands, for both female and male respondents (OR 1.41 CI 1.25–1.60 and OR 1.55 CI 1.25–1.93, respectively).

Students in a situation defined as being in a high strain study environment (high demands and low control over one’s studies) had almost double the odds of experiencing sexual harassment at Lund University than students in a comfortable study environment (low demands, high control) (OR 1.76, CI 1.48–2.10 for females and OR 2.06 CI 1.51–2.81 for males).

Experiencing low support from teachers and supervisors was significantly associated with exposure to sexual harassment among female students when compared to those experiencing high support (OR 1.36 CI 1.18–1.56). This association was not significant among male students. With regard to student support, odds for the association with sexual harassment were not significant for females or males.

Table 3 shows the results of the multivariable logistic regression analysis. In this analysis study strain (the combination of high demands and low control) is the exposure, and exposure to sexual harassment during one’s studies at Lund University is the outcome. The crude association between study strain and exposure to sexual harassment is shown, followed by three models adjusted for (1) age, (2) age and status as international student and parents’ country of birth and (3) age, status as international student, parents’ country of birth and support from lecturers and fellow students. All results are stratified by gender.

**Table 3.** Multivariable regression showing the adjusted association between study strain and exposure to sexual harassment among students at Lund University (*N* = 8897). Covariates have been added in three clusters, with age, background variables, and support variables, respectively. Odds ratios (OR) and 95% confidence intervals (CI).

	Crude		Model 1		Model 2		Model 3	
	Female	Male	Female	Male	Female	Male	Female	Male
**Study strain**								
High strain study environment	**1.76** (1.48–2.10)	**2.06** (1.51–2.81)	**1.73** (1.45–2.07)	**2.08** (1.52–2.84)	**1.76** (1.47–2.10)	**2.06** (1.51–2.81)	**1.67** (1.38–2.01)	**1.98** (1.44–2.74)
**Age**								
Low (18–25)			**1.95** (1.66–2.29)	1.15 (0.88–1.51)	**1.92** (1.63–2.25)	1.14 (0.87–1.49)	**1.88** (1.60–2.21)	1.11 (0.85–1.46)
**International student**								
No					0.94 (0.73–1.21)	1.38 (0.88–2.16)	0.93 (0.72–1.20)	1.35 (0.86–2.12)
**Parents’ country of birth**								
≥1 parent born in Sweden					**1.46** (1.20–1.78)	0.91 (0.66–1.24)	**1.44** (1.18–1.75)	0.89 (0.65–1.23)
**Lecturer support**								
Low							**1.19** (1.02–1.40)	1.18 (0.90–1.55)
**Student support**								
Low							0.90 (0.79–1.03)	0.87 (0.69–1.10)

Model 1: Adjusted for age; Model 2: Adjusted for age, international student, and parents’ country of birth; Model 3: Adjusted for age, international student, parents’ country of birth, lecturer support, and student support. Bold font indicates statistical significance.

### Psychosocial study environment

The association between high strain study environment (the combination of high demands and low control) and exposure to sexual harassment found in the crude model remained significant even in the fully adjusted model for both females and males when compared to being in a comfortable study environment (Model 3 aOR 1.67, CI 1.38–2.01 and aOR 1.98, CI 1.44–2.74, respectively). It is perhaps notable that the background characteristics found in models 1 and 2 had relatively little effect on this association, and even controlling for support reduced this association only slightly.

Receiving low support from lecturers was associated with higher odds of exposure to sexual harassment in the fully adjusted model for females compared to receiving high support (aOR 1.19, CI 1.02–1.40), but not for males. Low support from students was not significantly associated with exposure to sexual harassment in either group.

### Individual background characteristics

For females, the odds of being exposed to sexual harassment were higher for those 25 and under, compared to those 26 and older, even in the fully adjusted model (aOR 1.88, CI 1.60–2.21), an association not significant among males. Although being an international student did not show any significant associations with exposure to sexual harassment for females or males, having at least one parent born in Sweden was associated with higher odds of being exposed to sexual harassment for females (aOR 1.44, CI 1.18–1.75) but not for males in any of the models.

To explore the proportion of exposure to sexual harassment that could be prevented by eliminating the high strain study environment, the logistic regression-based population attributable fraction (PAF) was calculated using Miettinen’s formula [31]. Using odds ratios from the fully adjusted multivariable logistic regression, PAF for females was 14.0%, and for males the corresponding PAF was 15.4%.

Synergy indexes (SI) according to Rothman [33] were calculated to examine any modification to the association between demands and control and exposure to sexual harassment, and whether support had a buffering effect on the association between high strain study environments and exposure to sexual harassment. Unadjusted odds ratios obtained through bivariate regression analyses were used for these calculations [34]. Table 4 shows the results of this analysis.

**Table 4.** Analysis of effect modification between demands and control, as well as study strain and support and exposure to sexual harassment in a sample of university students from Sweden, presented as unadjusted odds ratios (or) with 95% confidence intervals (Ci) and synergy index (SI).

	Sexual harassment				
	N	% Cases	OR	95% CI	SI
**Demands & control**					
Comfortable (Low demands High control) (Ref)	300	16.0	1		
Passive (Low demands Low control)	427	22.8	1.32	1.12–1.56	
Active (High demands, High control)	454	24.3	1.49	1.27–1.76	
High strain (High demands, Low control)	689	36.8	2.00	1.72–2.32	1.2
**Supervisor/lecturer support**					
Low strain High support (Ref)	344	16.6	1		
Low strain Low support	837	19.8	1.25	1.09–1.43	
High Strain High support	67	22.3	1.44	1.07–1.94	
High strain Low support	622	27.1	1.88	1.62–2.18	1.3
**Student support**					
Low strain High support (Ref)	497	19.8	1		
Low strain Low support	684	18.1	0.89	0.78–1.02	
High Strain High support	248	28.8	1.64	1.37–1.96	
High strain Low support	441	25.4	1.38	1.20–1.60	0.7

SI > 1 can signify a synergistic (positive) effect modification [33]. Table 4 indicates a small synergistic effect between high demands, low control, and exposure to sexual harassment (SI 1.2). The same can be seen for support from Supervisors/Lecturers, high study strain environments, and exposure to sexual harassment (SI 1.3).

Synergy Index <1 can indicate an antagonistic (negative) effect modification. The interaction between support from students, high study strain, and exposure to sexual harassment showed a small antagonistic interaction (SI 0.7).

## Discussion

The results of this study show that experiencing high demands and low control in the study environment were independently associated with exposure to sexual harassment for both females and males.

As the first study to examine associations between the psychosocial university study environment defined by the DCS-instrument and sexual harassment, the fully adjusted model suggests that being in a study environment marked by high strain (defined as high demands and low control) is significantly associated with being exposed to sexual harassment for both females and males, although the association is stronger among males. Receiving low support from lecturers was also shown to be associated with higher odds of exposure to sexual harassment for females but not for males, and low support from students was not significantly associated with exposure to sexual harassment in either group. Evidence was found for a small synergistic effect between demands and control, and between supervisor support and high study strain regarding sexual harassment. A small antagonistic effect was found between high study strain and student support regarding exposure to sexual harassment. Population Attributable Fraction calculations suggest that 14% of sexual harassment among females, and 15% among males could be prevented by eliminating high strain study environments. Among individual characteristics, the odds of being exposed to sexual harassment at Lund University were significantly higher for females vs. males, for the younger age vs. older group and for those with at least one parent born in Sweden vs. those with both parents born abroad, respectively.

### Individual characteristics and sexual harassment

Many of the results of the bivariate analysis align with other studies of sexual harassment among university students. This includes the tendency for females to experience a higher exposure to sexual harassment than males in university settings [7], and a higher reporting rate of sexual harassment in the Nordic countries despite relatively high gender equality [35]. A more complete discussion of these individual factors is presented in Agardh et al. [9].

### Study environment and sexual harassment

Existing research on sexual harassment offers a variety of explanations as to how and why it continues to occur so broadly, ranging from the embeddedness of these behaviours in broader gender disparities [36] through theories of legal consciousness [37], and organisational perspectives [38]. This study posits that the occurrence of behaviours such as sexual harassment is in part based on workplace organisational factors and workplace social support. This is supported by the ‘Work environment hypothesis’ that proposes that poor social work environment, as defined by psychosocial work characteristics, may foster bullying and harassment in the workplace [39]. The lower OR reported by females in our study regarding the impact of study strain on SH could therefore be a reflection of the complexity of the issue of SH and its intersecting causes. Understanding these issues would require additional research.

Sexual harassment is sometimes considered to be on a continuum with other forms of harassment [40], and often occurs in workplaces and study environments with other forms of bullying and harassment [41]. Thus, it is reasonable to discuss the results of this study in relation to research in this area. Research into bullying and work environment according to the Demand-Control-Support model is a well-established field. This research has employed the DCS model on several different populations to examine the association between bullying and high demands, low control, and the potentially buffering effect of support. One could speculate that a high strain study environment is prone to foster factors such as high competitiveness and a general hierarchical set of attitudes and practices among students, which represents an organizational environment where specifically SH has been specifically proposed to be one strategy to maintain traditional gender power relations [3].

In this study, we found evidence of a positive association between high demands, and low control independently, and exposure to sexual harassment. This result is comparable to those of research conducted among police officers in Australia that found a positive association between high demands, low control, and bullying [20]. Similar findings were also found in research among blue collar workers in Belgium and Spain where a positive association was shown between demands and bullying, and a negative association between control and bullying [21].

Evidence in this study for an effect of study strain (high demands and low control) on exposure to sexual harassment is also supported in other research on bullying. In the study among blue collar workers in Belgium and Spain this relationship between strain and bullying is one of the most significant findings [21], and research conducted in the USA indicated a positive correlation between workplace bullying and job strain (as defined by high demands and low control), which appeared to be exacerbated by less supervisor and co-worker support, in an environment termed the ‘Boiler room environment’ [22]. This result of a strong buffering effect modification of support on the interaction between demands and control and bullying in the ‘Boiler room environment’ [22] was only partially supported in the current study where support from fellow students had a small buffering effect, but support from supervisors showed the opposite association. This finding has partially been echoed in research conducted among government employees in Sweden where perceived co-worker support was found to moderate the effects of bullying but not perceived supervisor support [42]. One possible explanation for this difference could be related to reverse causality in the study whereby those exposed to sexual harassment have sought and received support from supervisors or lecturers (see methodological considerations).

## Methodological considerations

This study’s strengths are the large size of the study population, and the engagement with students and student organisations in developing a survey applicable for a broad range of students.

As this is a cross-sectional study, the direction of causality between characteristics of the study environment and sexual harassment is not possible to ascertain. The association could be such that settings defined by high demands, low control, and low support give rise to conditions that may develop into sexual harassment as discussed in this study. The opposite could also be the case, however, such that those who have been exposed to sexual harassment experience a worse study environment in terms of demands, control, and support. Determining that there is an association between high strain study environments and sexual harassment is the first step in examining this relationship. Qualitative exploration, or longitudinal research would then be required to understand the temporality of this association and to better hypothesise about causality.

The survey had a response rate of around 32%. This rate is relatively low but in line with previous survey studies. A comparison between the study population and target population was conducted that showed no striking differences between the two groups [9]. However, no information was available about the non-respondents with regard to the exposure of interest and thus, there is the possibility of self-selection bias.

Some evidence exists that those who have experience of the topic in a survey are more likely to reply [43] and this could lead to overreporting of sexual harassment in the responses, albeit not necessarily to a change in the measured associations. The prominence of the #metoo campaign in the media at the time of this survey could have led to social desirability bias. Evidence suggest, however, that this can be minimised through self-reported surveys [44], and other research on sexual violence in university settings, supports the argument that this self-selection has limited effect on survey results [45].

The survey instrument did not collect data on sexual orientation and exposure to sexual harassment. Other research conducted in this area has highlighted that members of the LGBTQI+ community are often at higher risk of sexual harassment [46]. In addition, due to the small size of the group who identified as neither male nor female, we were also unable to conduct further analyses on this group. This highlights the need for additional research into psychosocial study environment and exposure to sexual harassment for the LGBTQI+ community members.

## Conclusions

Working to reduce situations of high strain study environments could be an effective strategy for reducing sexual harassment in university settings. Improving support from lecturers could also modify this relationship, but more research is required to identify causal pathways underlying this result.

## Data Availability

Data cannot be shared publicly because of the sensitive nature. Data are available from Lund University (contact via the correspondent author) for researchers who meet the criteria for access to confidential data.

## Paper context

Research shows that the work environment is an important factor for understanding sexual harassment. No research exists examining the associations between psychosocial study environment and sexual harassment in universities. This article examines this association. Results show that high study strain (combination of high demands and low control) was associated with exposure to sexual harassment among both female and male respondents. Reducing high strain environments could be an effective strategy for addressing sexual harassment in universities.
